# Digitally Scanned Radiographs versus Conventional Films for Determining Clarity of Periapical Lesions and Quality of Root Canal Treatment

**DOI:** 10.1155/2017/2427060

**Published:** 2017-11-15

**Authors:** Kholod Almanei, Rakan Alsulaimani, Sarah Alfadda, Sarah Albabtain, Reem Alsulaimani

**Affiliations:** ^1^Department of Restorative Dental Science, College of Dentistry, King Saud University, Riyadh, Saudi Arabia; ^2^College of Dentistry, King Saud University, Riyadh, Saudi Arabia; ^3^King Fahad Medical City, Riyadh, Saudi Arabia; ^4^Ministry of Health, Riyadh, Saudi Arabia

## Abstract

**Aim:**

To compare digital images of conventional radiographs with the original radiographs for perceived clarity of periapical lesions and the quality of root canal treatment.

**Materials and Methods:**

One hundred and four intraoral periapical radiographs of patients with endodontically treated teeth were randomly selected. The radiographs were digitized using an MD300 USB X-ray Reader. The digital images were transferred to an HP laptop. Three evaluators compared each conventional radiograph with the matching digital image. The images were ranked for clarity and assessed for diagnostic quality; data were analyzed using the Reliability Calculation “ReCal.”* Results*. Both the digital images and conventional films had comparable clarity and diagnostic quality. Results indicated a moderate agreement between the evaluators.

**Conclusions:**

Conventional radiographs digitized using an MD300 USB X-ray Reader have similar clarity and diagnostic quality in comparison to the original radiographs.

## 1. Introduction

Radiographs play an essential role in all phases of endodontic therapy: diagnosis, treatment, and postoperative evaluation/or follow-up. Periapical radiographs are the main intraoral radiographs used in endodontics, and these radiograph films have been utilized in root canal treatment for more than a decade [1]. The films must be exposed to an X-ray radiation source and then be chemically processed to produce images, which are conventional film-based radiographs. However, with the evolution in clinical dentistry, digital radiography has been introduced to overcome some drawbacks of conventional radiographs [2].

Digital radiography produces a digitized image that can be manipulated by a computer and displayed on-screen. Digitized images can be obtained either directly, by intraoral sensor or charge-coupled device, or indirectly, by scanning the conventional radiographs and transferring them to the computer (indirect digital imaging). One major advantage of a scanned digital image over a conventional radiographic film is that the scanned image can be manipulated for optimum diagnostic value [3]. This image also promotes further image enhancement with a wide array of tools, density and contrast alteration, gray scale inversion, magnification, pseudocolor, and pseudo-3D [4]. In addition, it aids in patient instruction and in the patient's acceptance of the treatment [5]. A digitally scanned image can also be transmitted electronically and stored in patient records for proper documentation and easier retrievability [6].

There are several methods of radiographic digitization and scanning, including using video capture, a digital camera, a hard scanner, or a flatbed scanner [6]. Recently, a specialized scanner called a dental X-ray film reader has been used to convert dental X-ray films into digital images. These scanned images can be transferred from the film reader to a personal computer through a universal serial bus (USB) cable.

Radiographs with a high degree of clarity aid endodontists in determining the quality of root canal therapy and the presence or absence of periapical lesions [7]. Although digital radiography holds several advantages over conventional radiographs, studies have revealed that digital radiographs are similar in interpretive quality to conventional films [8, 9]. On the other hand, a few studies have demonstrated that digital camera images and scanned images did not produce images of diagnostic quality [3, 5]. However, no study showed the differences between conventional films and the digital images of scanned conventional radiographs from a dental X-ray film reader. Therefore, the purpose of this study is to compare periapical radiographs digitally scanned by a dental X-ray reader with original conventional films for differences in perceived clarity of periapical lesions and the quality of root canal treatment.

## 2. Materials and Methods

A total of 104 postoperative periapical radiographs from the undergraduate endodontic clinic of the dental school of King Saud University were used for the study. These radiographs depicted images of endodontic treatment of single- and multirooted teeth. The radiographs were obtained using size 1 (for anterior teeth) and size 2 (for posterior teeth) type E radiographic film (EktaspeedPlus, Kodak Company, Rochester, NY, USA) in an X-ray unit (Siemens Heliodent “DS” X-ray, Germany) at 7 mA and 60 kVp following a paralleling technique. The radiographs were processed using an automatic processor (DENT-X 9000/810 Basic Processors, NY, USA).

**Figure 1:**
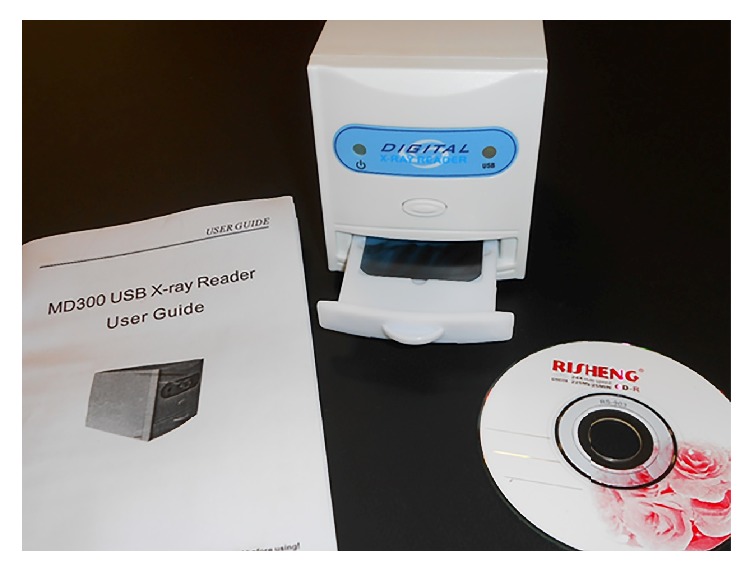
MD300 USB X-ray Reader.

**Figure 2:**
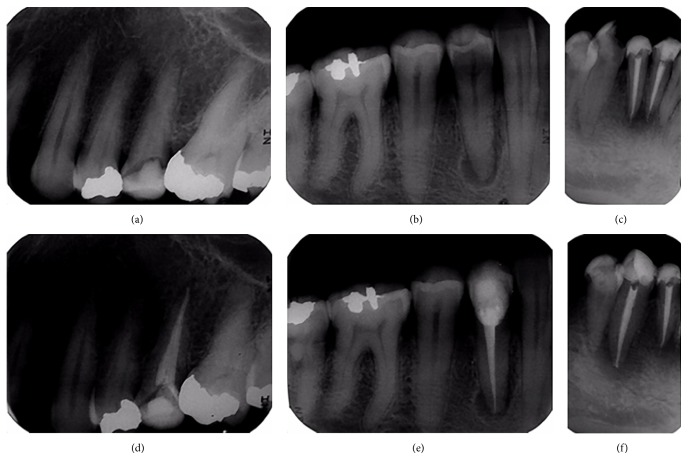
Digitized images of preoperative (a, b, c) and postoperative (d, e, f) conventional periapical radiographs using the MD300 USB X-ray Reader.

All the periapical radiographs were digitized using the MD300 USB X-ray Reader (Risheng, China) (Figure 1). The digital images were immediately transferred to a computer (HP Pavilion g6 Notebook PC with 15.6-inch LED monitor, with a screen resolution of 1366 × 768) using a USB cable. All images were saved as JPEG files (width 480 pixels, height 640 pixels, and horizontal and vertical resolution 96 dpi) (Figure 2).

Three calibrated evaluators (an undergraduate dental student, a general dental practitioner, and an endodontist) compared the digital images created from the X-ray reader with the conventional films. The conventional radiographs were examined on a viewing box without magnification, while the digital images were viewed on a laptop monitor without magnification or zooming. The two variables evaluated were the clarity of periapical lesions and the quality of root canal treatment. If the digital image showed a greater visibility of periapical lesions and misshapes than did the conventional radiograph, it was scored as positive (+). If the digital image exhibited less visibility of periapical lesions and misshapes than did the conventional radiograph, it was scored as negative (−). Lastly, if the images were equal in their visibility of periapical lesions and misshapes, they were scored as equal (=). The adequacy of obturation, length, and density was also evaluated as previously described [10]. If the digital image showed more inadequacy in length or density than did the conventional radiograph, it was scored as positive (+). If the digital image revealed more adequacy in length or density than did the conventional radiograph, it was scored as negative (−). If the images were equal in their adequacy of length and density, they were scored as equal (=). The results were compared statistically. The kappa test was used to measure the level of agreement between the three evaluators.

## 3. Results

Table 1 presents evaluation results comparing digital images to conventional radiographs in determining the clarity of periapical lesions and the quality of root canal treatment. In order to examine the agreement in ratings between the evaluators on the comparison of the digital images and conventional film, kappa statistics were computed using the Reliability Calculator “ReCal” [11]. Cohen's kappa was calculated for the interrater, yielding a level of agreement between the two methods (digital and conventional radiographs) of *κ* = .635, which was considered a good level of agreement [12]. The percent of agreement between raters using digital versus conventional film was 82.9%. Fleiss's kappa was used to examine the agreement between raters; the results indicated moderate agreement (*κ* = .594) and an average pairwise agreement of 81.2% (Table 2). Overall, these findings suggest that both methods of assessment yield comparable results. Hence, there is a general sense of agreement among different raters using digital images and conventional films.

## 4. Discussion

The digitized radiograph image has distinct superiority over the conventional film [3]. Digitized images allow an easier picture archiving and communication system to be implemented [13]. Because of the technological possibilities available through digital software, digital images can enhance the conditions of dental diagnosis, treatment planning, and follow-up [14].

This study shows the differences between digitally scanned radiographs and conventional films in terms of the clarity of periapical lesions and the quality of root canal treatment. The results showed that the digital images and conventional films had comparable clarity and diagnostic quality.

Several studies have been conducted to explore the quality of digitized radiographs in comparison with their conventional counterparts; however, the results of these studies have been discordant [3]. Fuge et al. [3] compared digital images with conventional films for the clarity of the endodontic file in relation to the radiographic apex. They found that digitized images were inferior to conventional radiographs in determining the end point of size 6 K-files in molar root canals.

Goga et al. [5] evaluated the clarity and diagnostic quality of digitized radiographs compared with conventional radiographs. The result of their study indicated that digitized periapical radiographs did not improve the clarity and diagnostic quality in comparison to conventional radiographs. However, our findings showed a similarity between digitally scanned images and conventional films in terms of clarity and diagnostic quality. This controversy could be due to the use of different scanner tools in the digitization of radiographs. In the present study, the MD300 USB X-ray Reader (Risheng, China) was used to digitize the periapical conventional films; this reader enlarges the X-ray film by up to 50 times. With this piece of equipment, X-rays can be converted to digital images and transmitted to computers immediately through a USB cable. This reader can read any standard dental X-ray film and adjust the image's contrast, brightness, and color. Images can be treated to be blurred, sharpened, reversed, and falsely colored. The reader can also transfer correlative data onto a storage device.

On the other hand, Schmitd et al. [4] studied the radiographic measurements obtained with conventional and indirect digital imaging during endodontic treatment. They concluded that the quality of scanned digital images was superior to that of original conventional films. Similarly, Malleshi et al. [15] analyzed the clarity and diagnostic value of digital images in comparison with conventional intraoral radiographs. They demonstrated that digitized images resulted in enhanced image clarity and improved diagnostic quality; however, their findings were not substantiated by the present study. This contradiction could be attributed to the use of multiple types of software in which digitally scanned images can be adjusted to variable brightness and contrast.

For the present study, intraexaminer agreement was good; however, the interexaminer agreement was fair to good. This semi-low agreement among the examiners can be explained by the difference in years of experience among the examiners.

In conclusion, digitizing conventional dental radiographs using the MD300 USB X-ray Reader produced images with the same clarity and diagnostic quality of conventional radiographs. Based on these results, the MD300 USB X-ray Reader seems to be an acceptable tool with which to digitize conventional films. However, further studies on the enhancement tools of scanning X-ray systems are required to maximize the benefit of X-ray digitization.

## Figures and Tables

**Table 1:** Evaluation scores comparing digital images to conventional radiographs by evaluator.

	Examiner 1		Examiner 2		Examiner 3	
	*n*	%	*n*	%	*n*	%
Presence of misshapes						
−	4	3.8	6	5.8	13	12.5
+	5	4.8	4	3.8	10	9.6
=	95	91.3	94	90.4	81	77.9
Obturation density						
−	5	4.8	8	7.7	6	5.8
+	9	8.7	20	19.2	11	10.6
=	90	86.5	76	73.1	87	83.7
Obturation length						
−	2	1.9	9	8.7	8	7.7
+	10	9.6	20	19.2	9	8.7
=	92	88.5	75	72.1	86	82.7
Periapical lesion						
−	9	8.7	13	12.5	12	11.5
+	4	3.8	10	9.6	7	6.7
=	91	87.5	81	77.9	85	81.7

*Note.* When the evaluator determined that the digital image showed greater detail than the radiograph a “+” was scored, if they were equal a “=” was scored, and if the detail was less a “−” was scored.

**Table 2:** Summary of results.

Comparison	Kappa	Average pairwise agreement
Interrater	.634	82.9
Intrarater	.594	81.2
